# Result of Abortion on Milk

**Published:** 1896-04

**Authors:** 


RESULT OF ABORTION UPON THE MILK.[1] Not infrequently farmers and milk-dealers, when detected in selling adulterated milk, resort to the subterfuge of explaining the condition as being due to a modification of the secretion brought about by abortion in cows. A scientific substantiation of this explanation has not been attained. The authors made thorough examination in seven different cows as to the result of abortion upon the constitution of the milk-secretion, and came to the conclusion that the results would not warrant such an assumption. They found, indeed, a variation of from 2.37 to 5.75 in the fat. This, however, was easily accounted for by the individuality of the separate cases, and the other elements were in proportion to the fat. They also examined the milk of one cow suffering from placenta-retentia, which likewise failed to give noticeable change in its constitution.—SHAFFER and HESS, *in Zeitschr. for Meat and Milk Hygiene, Berlin.*

[1, 2] Translated for the Journal by Frank H. Miller, V.S., Royal Veterinary University, Berlin, Germany.

